# Elucidation of HHEX in pancreatic endoderm differentiation using a human iPSC differentiation model

**DOI:** 10.1038/s41598-023-35875-1

**Published:** 2023-05-29

**Authors:** Ryo Ito, Azuma Kimura, Yurie Hirose, Yu Hatano, Atsushi Mima, Shin-Ichi Mae, Yamato Keidai, Toshihiro Nakamura, Junji Fujikura, Yohei Nishi, Akira Ohta, Taro Toyoda, Nobuya Inagaki, Kenji Osafune

**Affiliations:** 1 Center for iPS Cell Research and Application (CiRA), Kyoto University, 53 Kawahara-cho, Shogoin, Sakyo-ku, Kyoto, 606-8507 Japan; 2 Department of Diabetes, Endocrinology and Nutrition, Graduate School of Medicine, Kyoto University, 54 Kawahara-cho, Shogoin, Sakyo-ku, Kyoto, 606-8507 Japan

**Keywords:** Developmental biology, Stem cells, Endocrinology

## Abstract

For pluripotent stem cell (PSC)-based regenerative therapy against diabetes, the differentiation efficiency to pancreatic lineage cells needs to be improved based on the mechanistic understanding of pancreatic differentiation. Here, we aimed to elucidate the molecular mechanisms underlying pancreatic endoderm differentiation by searching for factors that regulate a crucial pancreatic endoderm marker gene, NKX6.1. Unbiasedly screening an siRNA knockdown library, we identified a candidate transcription factor, HHEX. *HHEX* knockdown suppressed the expression of another pancreatic endoderm marker gene, *PTF1A*, as well as *NKX6.1*, independently of *PDX1*, a known regulator of *NKX6.1* expression. In contrast, the overexpression of *HHEX* upregulated the expressions of *NKX6.1* and *PTF1A*. RNA-seq analysis showed decreased expressions of several genes related to pancreatic development, such as *NKX6.1*, *PTF1A*, *ONECUT1* and *ONECUT3*, in *HHEX* knockdown pancreatic endoderm. These results suggest that HHEX plays a key role in pancreatic endoderm differentiation.

## Introduction

Type 1 diabetes is a condition in which pancreatic β cells are destroyed by autoimmunity, resulting in an absolute deficiency of insulin^1^. Although insulin injection is the standard treatment, the complete return to normal blood glucose maintenance is often difficult, resulting in hypoglycemia and hyperglycemia. Whole-organ pancreas and islet transplantations are among the treatments that may achieve normoglycemia, but these therapies are hampered by severe donor shortages^1–3^. Since pluripotent stem cells (PSCs), such as embryonic stem cells (ESCs) and induced pluripotent stem cells (iPSCs), have infinite proliferative and pluripotent differentiation potential, they are expected sources of pancreatic β cells for regenerative medicine. Recently, in vitro differentiation methods from human PSCs to pancreatic β cells have substantially advanced^4–10^. However, detailed elucidation of the differentiation mechanism is required to achieve higher differentiation efficiency.

During the developmental process of endoderm lineages, the posterior foregut expresses a specific marker gene called pancreatic and duodenal homeobox 1 (PDX1) and differentiates into the pancreas, duodenum, extrahepatic bile duct and posterior stomach^11^. A part of the posterior foregut differentiates into pancreatic endoderm which expresses NK6 homeobox 1 (NKX6.1) and pancreas associated transcription factor 1a (PTF1A). These PDX1^+^NKX6.1^+^PTF1A^+^ pancreatic endoderm cells act as multipotent pancreatic progenitor cells that give rise to all pancreatic lineages^12^. PDX1^+^NKX6.1^+/high^ pancreatic endoderm cells derived from human PSCs mature into functional β cells and improve hyperglycemia when transplanted into immunodeficient diabetes model mice, whereas human PSC-derived PDX1^+^NKX6.1^+/low^ cells differentiate into polyhormonal cells and do not functionally mature^13^. Thus, the expression of NKX6.1 in pancreatic endoderm may play a crucial role in the subsequent differentiation to pancreatic β cells. It is known that Pdx1 promotes Nkx6.1 expression in murine pancreatic endoderm and that in E10.5 *Pdx1*^*−/−*^ mice, the expression of Nkx6.1 is absent from the residual pancreatic epithelium^14^. However, regulators of NKX6.1 expression other than PDX1 in pancreatic endoderm have not been fully elucidated.

PSCs are powerful tools for elucidating developmental mechanisms when used in in vitro differentiation systems to mimic developmental processes in embryos^9,10,15^. In the present study, we aimed to identify regulatory genes and elucidate their roles in pancreatic endoderm differentiation using a human iPSC differentiation model. We established a screening system using an siRNA knockdown library and found that the transcription factor hematopoietically expressed homeobox (HHEX) is a key regulator for pancreatic endoderm differentiation. We found that HHEX affects several genes related to pancreatic development and is essential for pancreatic endoderm differentiation.

## Results

### Development of siRNA screening system for regulators of NKX6.1 expression in pancreatic endoderm

**Figure 1:**
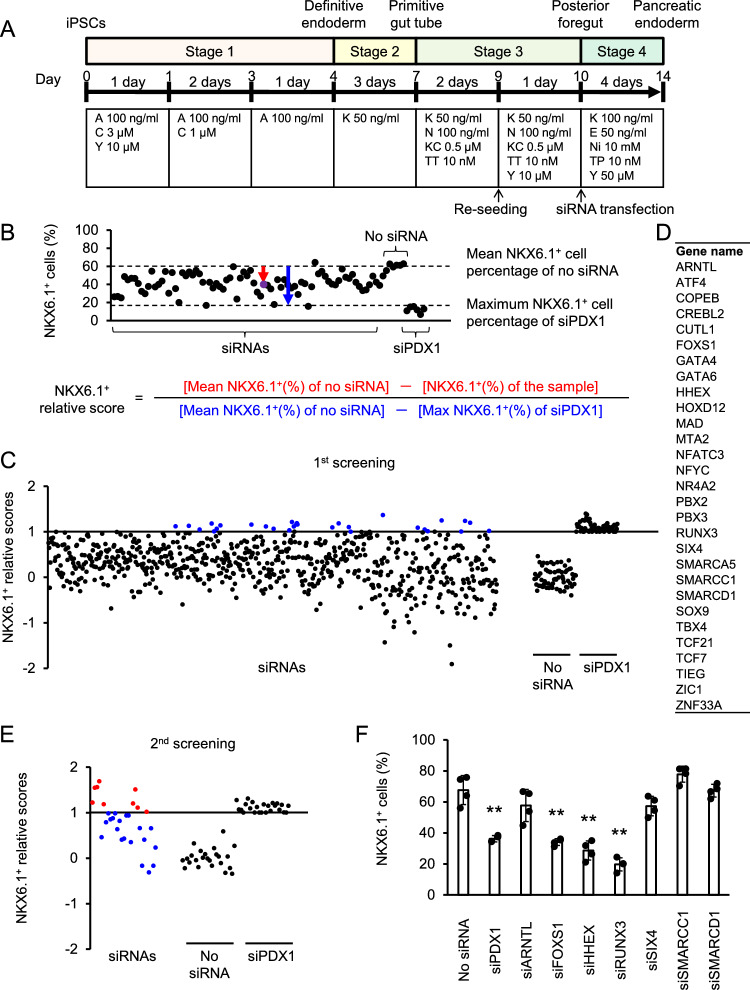
Screening the siRNA knockdown library for regulators of NKX6.1 expression in pancreatic endoderm. **(A)** A schematic diagram of the procedures used to differentiate pancreatic endoderm cells from iPSCs. *A* activin A; *C* CHIR99021; *Y* Y-27632; *K* KGF; *N* Noggin; *KC* KAAD-cyclopamine; *TT* TTNPB; *E* EGF; *Ni* nicotinamide; *TP* TPB. **(B)** A schematic diagram showing NKX6.1^+^ relative scores for each gene in the screening. **(C)** A scatterplot showing NKX6.1^+^ relative scores of each siRNA from the 1st screen. **(D)** The list of 29 hit genes in the 1st screening. **(E)** A scatterplot showing NKX6.1^+^ relative scores of each siRNA from the 2nd screen. **(F)** Validation of 7 hit genes using siRNAs with different sequences. The data from four independent experiments are presented as the mean ± SD (n = 4). **p < 0.01 by one-way ANOVA with Dunnett’s test for comparison with no siRNA.

To identify the regulators of NKX6.1 expression in human pancreatic endoderm, we established a screening system using siRNA-mediated gene knockdown. As shown in Fig. 1A, we differentiated human iPSCs into pancreatic endoderm by modifying our previously reported induction method using a two-dimensional monolayer culture^10^. Cell re-seeding was performed on Stage 3 day 2 (day 9), which is the differentiation stage of posterior foregut. The next day (day 10), siRNA transfection was performed when changing to Stage 4 medium. The estimated value of the siRNA transfection efficiency using fluorescent RNA oligo was nearly 100% (Fig. S1A). We used siRNA against the *PDX1* gene (siPDX1) as a positive control, since this gene is known to promote the expression of NKX6.1^14^, and found siRNAs at more than 7.5 nM are effective (Fig. S1B). The effect of each siRNA on NKX6.1 expression was evaluated by measuring the percentage of NKX6.1^+^ cells among total cells in immunostaining images. The percentage of NKX6.1^+^ cells was similar among different wells and decreased with the transfection of siPDX1 (Fig. S1C). Thus, we developed a screening system for regulators of NKX6.1 expression in human pancreatic endoderm using siRNA-mediated gene knockdown.

### siRNA screening identified HHEX as a regulator of NKX6.1 expression

We searched for NKX6.1 regulators by screening siRNAs against 719 available transcription factors extracted from the FANTOM5 (Function ANnoTation Of the Mammalian genome-5) database (fantom.gsc.riken.jp/5). We calculated the NKX6.1^+^ relative score by dividing the difference between the mean NKX6.1^+^ cell percentage (%) of the negative controls without siRNAs and the NKX6.1^+^ cell percentage (%) with the tested siRNAs by the difference between the mean NKX6.1^+^ cell percentage (%) of the negative controls without siRNAs and the maximum NKX6.1^+^ cell percentage (%) of the positive controls with siRNA against PDX1 (Fig. 1B). We defined genes with an NKX6.1^+^ relative score greater than 1 as a hit. Consequently, 29 hit genes were identified by the primary screening (blue dots; Fig. 1C, D). A secondary screening was performed using these 29 hit genes in the same manner as the primary screening, narrowing down the hits to nine genes (red dots; Fig. 1E), among which two genes (GATA4 and GATA6) were previously reported as regulators of PDX1 expression and therefore excluded^16^. Reproducibility was confirmed with three of the remaining seven genes by examining siRNAs against other sequences in the same seven genes (Fig. 1F). We further excluded two of the three genes; *FOXS1*, whose knockdown only modestly decreased the NKX6.1^+^ cell percentage according to a flow cytometry analysis (Fig. S2A), and *RUNX3*, whose knockdown caused a decrease in the number of PDX1^+^ cells and total cells as well as NKX6.1^+^ cells, probably due to non-specific cytotoxicity (Fig. S2B). Finally, *HHEX* was extracted as a candidate.

### HHEX is essential for pancreatic endoderm differentiation

**Figure 2:**
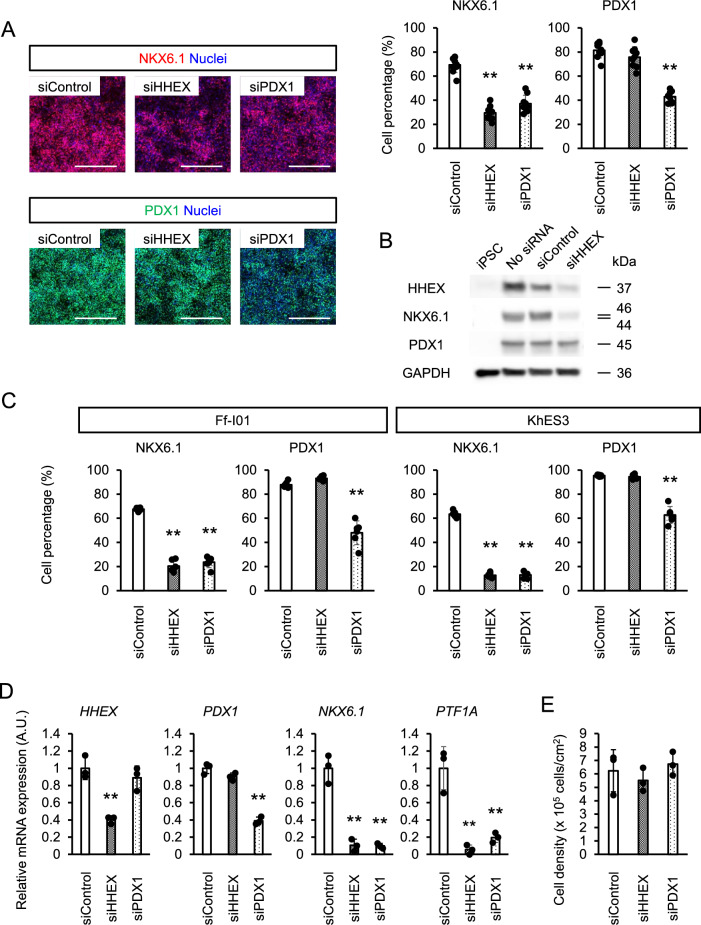
The effects of *HHEX* knockdown on pancreatic endoderm cells. **(A)** Immunofluorescence images (left panels) and percentage (right panels) of NKX6.1^+^ and PDX1^+^ cells at Stage 4 upon knockdown of *HHEX* or *PDX1*. Scale bars; 500 µm. **(B)** The protein expression of HHEX, NKX6.1 and PDX1 upon *HHEX* knockdown at Stage 4 by western blotting. **(C)** Percentage of NKX6.1^+^ and PDX1^+^ cells at Stage 4 upon knockdown of *HHEX* or *PDX1* in other PSC lines. **(D)** Expressions of *HHEX*, *PDX1*, *NKX6.1* and *PTF1A* at Stage 4 upon knockdown of *HHEX* or *PDX1*. **(E)** The number of Stage 4 cells upon knockdown of *HHEX* or *PDX1*. The data from three independent experiments are presented as the mean ± SD (n = 9 for A; n = 6 for C; n = 3 for D and E). **p < 0.01 by one-way ANOVA with Dunnett’s test for comparison with siControl.

We further verified *HHEX* knockdown in pancreatic endoderm (Fig. 2A, B). The analysis revealed that *HHEX* knockdown reduced both the NKX6.1^+^ cell percentage and NKX6.1 protein expression, but not the PDX1^+^ cell percentage or PDX1 protein expression, which suggests that HHEX affects NKX6.1 expression by mechanisms independent of PDX1. The decrease in the NKX6.1^+^ cell percentage by *HHEX* knockdown was observed in the iPSC line 585A1, iPSC line Ff-I01 and human ESC line KhES-3 (Fig. 2C), indicating that the effect was not cell line-specific. Next, we evaluated the effects of *HHEX* knockdown on the expression of another pancreatic endoderm marker gene, *PTF1A*, and found that its expression too was reduced (Fig. 2D). We also considered whether HHEX promotes the proliferation of pancreatic endoderm cells, because cultures with high cell density facilitate the differentiation of pancreatic endoderm cells from hESCs/iPSCs^9^. However, *HHEX* knockdown, like *PDX1* knockdown, did not affect the total cell number (Fig. 2E). Therefore, we concluded that HHEX regulates NKX6.1^+^ pancreatic endoderm cell differentiation and not proliferation.

### HHEX contributes to the generation of pancreatic endoderm

**Figure 3:**
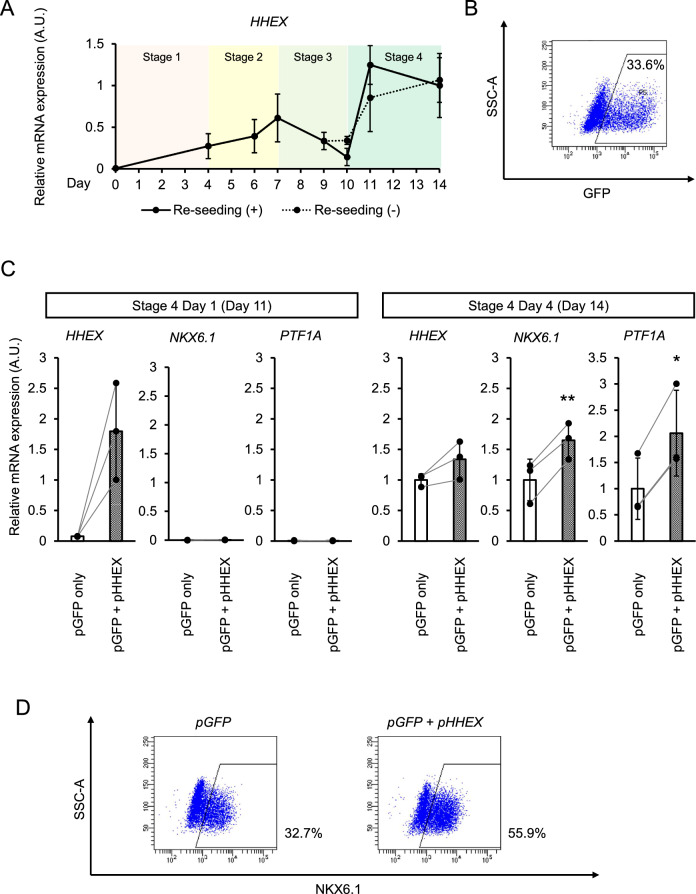
HHEX contributes to the generation of pancreatic endoderm. **(A)** The expression of *HHEX* during differentiation from the undifferentiated state to pancreatic endoderm cells with (solid line) or without re-seeding on Stage 3 day 2 (dotted line). **(B)** A representative flow cytometer plot showing the transfection efficiency of *pCXLE-eGFP* into pancreatic endoderm cells by electroporation. **(C)** Expressions of *HHEX*, *NKX6.1* and *PTF1A* in Stage 4 day 1 (day 11; left panels) and Stage 4 day 4 (day 14; right panels) cells overexpressing *HHEX* on Stage 4 day 0 (day 10). **(D)** Representative flow cytometer plots showing NKX6.1^+^ cell percentage upon the overexpression of *HHEX*. The data from three independent experiments are presented as the mean ± SD (n = 3). *p < 0.05 and **p < 0.01 by two-tailed Student's t-test.

HHEX is expressed in endoderm lineage tissues at early developmental stages^17,18^. We therefore examined the temporal expression of *HHEX* in our iPSC differentiation culture system to compare the expression pattern with embryos. In our culture, *HHEX* expression was increased through day 4 of the stage of the definitive endoderm (Stage 1) to day 3 of the primitive gut tube (Stage 2) (day 7), decreased by day 3 of the posterior foregut (Stage 3) (day 10), and then substantially increased again by day 1 of the pancreatic endoderm (Stage 4) (day 11) (Fig. 3A). This increase at Stage 4 was observed regardless of re-seeding on Stage 3 day 2 (day 9) (dotted line; Fig. 3A). To examine whether the increase at Stage 4 promotes the expressions of *NKX6.1* and *PTF1A*, we overexpressed *HHEX*. The cell transfection efficiency of *pCXLE-eGFP* using electroporation was about 30% (Fig. 3B). When we modified the Stage 4 culture by removing EGF, nicotinamide and TPB from the original Stage 4 medium, the rise of *HHEX* expression was delayed (Fig. S3A) compared to the early and rapid increase in *HHEX* expression in the original Stage 4 cultures (Fig. 3A). In the modified conditions, *HHEX* was overexpressed by the transfection of *pCXLE-HHEX*. As a result, *HHEX* overexpression restored the *HHEX* upregulation in early Stage 4 (day 11) (Fig. 3C). The expression of *NKX6.1* and *PTF1A* also increased three days after *HHEX* upregulation (day 14) (Fig. 3C). In addition, *HHEX* overexpression increased the NKX6.1^+^ cell percentage (Fig. 3D).

### HHEX is involved in pancreatic endoderm maturation

**Figure 4:**
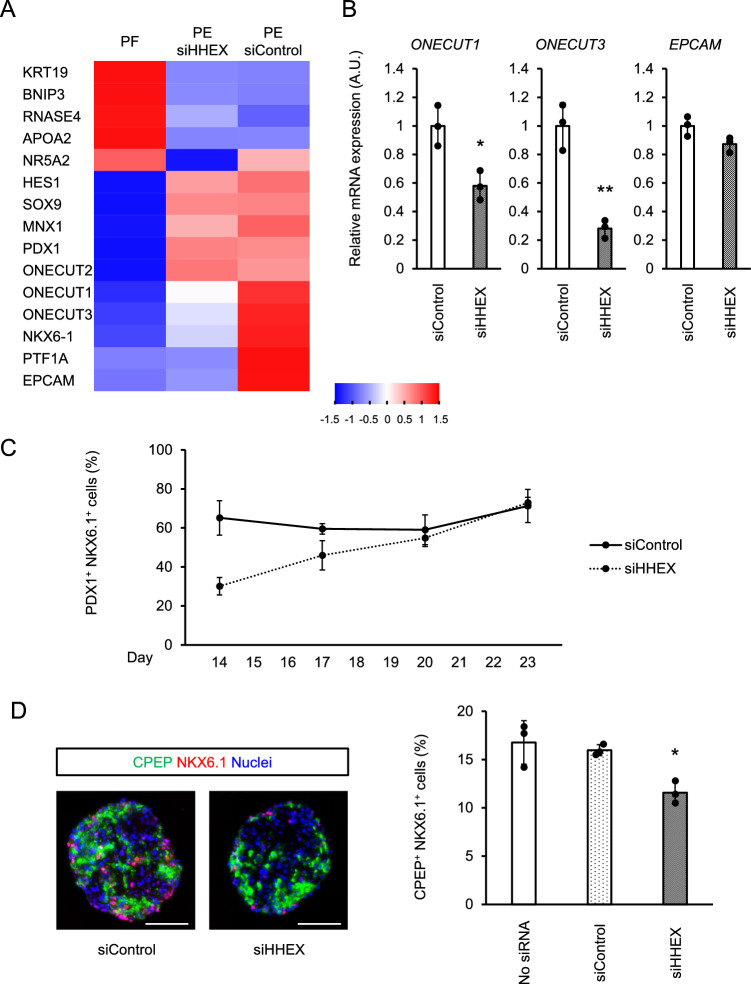
*HHEX* knockdown results in defective pancreatic endoderm generation. **(A)** A heatmap representing normalized Z-scores of pancreatic marker expressions in posterior foregut (PF) and pancreatic endoderm (PE) with or without *HHEX* knockdown. **(B)** Expressions of *ONECUT1*, *ONECUT3* and *EPCAM* at Stage 4 with or without *HHEX* knockdown. **(C)** Temporal changes in the percentage of NKX6.1^+^PDX1^+^ cells under continued Stage 4 culture with (dotted line) or without *HHEX* knockdown (solid line). **(D)** Immunofluorescence images (left panels) and percentage (right panels) of CPEP^+^NKX6.1^+^ cells at Stage 6 with or without *HHEX* knockdown. Scale bars, 100 µm. The data from three independent experiments are presented as the mean ± SD (n = 3). *p < 0.05 and **p < 0.01 by two-tailed Student's t-test in (**B**) and by one-way ANOVA with Dunnett’s test for comparison with siControl in (**D**).

To verify the detailed roles of HHEX, the transcriptional profile of iPSC-derived pancreatic endoderm cells, in which *HHEX* was knocked down, was analyzed by RNA sequencing (RNA-seq; Fig. 4A). Gene expressions in the *HHEX*-knockdown pancreatic endoderm cells (PE siHHEX) were closer to control pancreatic endoderm cells (PE siControl) than posterior foregut cells (PF). Among the genes related to pancreatic development, the expressions of *ONECUT1*, *ONECUT3* and *EPCAM* as well as *NKX6.1* and *PTF1A* were downregulated by *HHEX* knockdown (Fig. 4A). Reproducibility was confirmed for *ONECUT1* and *ONECUT3* by multiple rounds of qRT-PCR (Fig. 4B). We also considered whether transient *HHEX* knockdown diverts cells from the pancreatic lineage and permanently impairs their ability to differentiate towards pancreatic endoderm but found, with continued culturing in Stage 4 medium, the percentage of PDX1^+^NKX6.1^+^ pancreatic endoderm cells in siHHEX gradually reached the level of siControl (Fig. 4C). Moreover, *HHEX* knockdown did not downregulate definitive endoderm-related genes or consistently upregulate hepatobiliary and duodenal development-related genes (Figure S3B). The differentiation of *HHEX*-knockdown pancreatic endoderm cells into C-peptide (CPEP)^+^NKX6.1^+^ pancreatic β cells after Stage 4 culture for 4 days had a modestly lower efficiency than the differentiation of controls (Fig. 4D). Therefore, we concluded that *HHEX* is essential for the differentiation of mature pancreatic endoderm cells that express *NKX6.1*, *PTF1A*, *ONECUT1* and *ONECUT3* and show the differentiation potential into β cells, but not for pancreatic lineage commitment itself.

In the RNA-seq data, the expression of *Sonic Hedgehog* (*SHH*) was upregulated in *HHEX*-knockdown pancreatic endoderm cells (data not shown). It has been reported that HHEX is involved in liver bud development through an inhibitory effect on SHH^19^. Given the possibility that HHEX is also involved in pancreatic endoderm differentiation through an inhibitory effect on SHH, we examined whether SHH signaling affected the expression of NKX6.1. However, the percentage of NKX6.1^+^ cells was independent of the concentration of SHH or a hedgehog signaling inhibitor, KAAD-Cyclopamine (Figure S3C). Therefore, the suppression of *SHH* expression by HHEX does not affect the differentiation of pancreatic endoderm cells.

## Discussion

In this study, a knockdown screening system using siRNA and a pancreatic lineage differentiation model from human iPSCs was constructed to elucidate the mechanisms of pancreatic endoderm differentiation. Recently, we and others have conducted comprehensive screening using RNA interference techniques in human iPSC culture systems to determine the mechanisms of action of the proliferation-promoting compound for PDX1^+^ pancreatic progenitor cells and the mechanisms of skin epithelial cell differentiation^20,21^. Here, we searched for NKX6.1 regulators in pancreatic endoderm differentiation using similar siRNA screening and found that the transcription factor HHEX is involved. These screening systems are highly versatile for elucidating the differentiation and proliferation mechanisms of various organs and tissues.

Hhex is expressed in endoderm lineage tissues early in mouse development (around embryonic day (E) 7.0). It is then expressed in the ventral-most cells of the anterior intestinal portal at E8.5 and of the ventral pancreas at E9.5^22–24^. The deletion of Hhex causes hypoplasia of the thyroid, thymus, lungs, liver and ventral pancreas^17,18,23,24^. The effects of Hhex deletion on the dorsal pancreas remain unknown, partly because Hhex-deficient mice are embryonic lethal around E15.0, making a detailed evaluation difficult^17^. However, *Hhex* expression starts in the dorsal pancreas of wild-type mice at around E10.0 to 11.5, and its expression becomes restricted to the pancreatic epithelia and duct by around E16.5 to 18.5^18,25^. Therefore, it is possible that Hhex plays roles in pancreatic development even in late embryonic stages. Our results revealed that HHEX regulates the expression of NKX6.1 and PTF1A in the differentiation of pancreatic endoderm.

Additionally, Hhex plays a crucial role in the differentiation of somatostatin-secreting δ cells in the pancreatic islets of adult mice^26^, suggesting HHEX may have different functions at multiple pancreatic developmental stages. The current study revealed a novel function of HHEX in pancreatic endoderm differentiation: *HHEX* knockdown in pancreatic endoderm modestly lowers the pancreatic β cell differentiation efficiency. Although HHEX is not expressed in adult human or mouse pancreatic β cells^26^, there is the possibility that lower HHEX expression during development leads to the loss of pancreatic endoderm cells, resulting in a decreased number of pancreatic β cells and an increased risk of developing diabetes. Indeed, a human genome-wide associated study (GWAS) identified the *HHEX* gene locus as substantially contributing to the susceptibility for type 2 diabetes^27^, and several variants in or near the *HHEX* gene are associated with the increased risk of type 2 diabetes^28,29^. Future studies on the function of HHEX at each pancreatic developmental stage may elucidate the mechanisms underlying the onset of type 2 diabetes and contribute to the development of novel therapeutic approaches.

The RNA-seq analysis in this study showed that *HHEX* knockdown reduced the expression of the pancreatic endoderm marker genes *NKX6.1* and *PTF1A* as well as other genes related to pancreatic development, including *ONECUT1* and *ONECUT3*. ONECUT1 was recently reported to bind to numerous active enhancers while co-binding with GATA6, PDX1, NKX6.1 and FOXA2 at posterior foregut and pancreatic endoderm stages in pancreatic development, indicating it is a crucial regulator for pancreatic endoderm differentiation^30^. Taken together with the results of the current study, HHEX may be involved in pancreatic endoderm differentiation by regulating *ONECUT1* expression. Onecut3 expression depends on the activation by Onecut1^31^, which is consistent with the expression patterns of *ONECUT1* and *ONECUT3* in our study. In contrast, Onecut2 is known to play partially redundant roles with Onecut1 in pancreas morphogenesis^32^, which might explain the different expression patterns of *ONECUT1* and *ONECUT2* in our study (Fig. 4A). Considering previously reported downstream factors of HHEX, the expression of *SHH*, for which HHEX is inhibitory in liver bud development^19^, was upregulated in *HHEX*-knockdown pancreatic endoderm cells. However, SHH did not affect pancreatic endoderm differentiation (Figure S3C). In addition, the expression of *EOMES* and *Flk-1/KDR*, which are downstream factors of HHEX in hepatic and hemangioblast development, respectively^33,34^, did not show significant changes in *HHEX*-knockdown pancreatic endoderm cells in our current study (data not shown). Future studies should consider the role of HHEX in the gene expression regulation network of pancreatic endoderm.

A more recent study reported that HHEX is involved in a commitment to pancreatic lineage differentiation^25^. Some findings in that study, including the effects of *HHEX* knockout on *NKX6.1* expression, are consistent with ours. However, *HHEX* knockout significantly affected *PDX1* expression in the previous study but not ours. Furthermore, the deletion of *HHEX* impaired pancreatic differentiation and caused ectopic liver differentiation, whereas our study did not show a preference for the hepatic differentiation of *HHEX*-knockdown cells. The possible reasons for these discrepancies include the stage of the *HHEX* deletion (undifferentiated ESCs versus conditional knockdown at the pancreatic endoderm stage). The advantage of our approach is that we elucidated more specific roles of HHEX in pancreatic endoderm, eliminating the effects of other stages from the undifferentiated state to posterior foregut.

Our current study has several limitations. First, since we have conducted knockdown experiments using siRNA, which do not completely knockout gene expression, the effects of remaining HHEX cannot be ruled out. Second, our study is in vitro and only used cultured cells; confirmational work in vivo about human pancreatic development is required. Finally, other molecular mechanisms involving HHEX, such as binding to promoter or enhancer regions and molecular interactions, cannot be inferred by our experiments.

In conclusion, using an siRNA screening system and human iPSCs, we revealed that the transcription factor HHEX is involved in pancreatic endoderm development by regulating the expression of the pancreatic development-related genes NKX6.1, PTF1A, ONECUT1 and ONECUT3. These findings should contribute to understanding the detailed mechanisms of pancreatic endoderm development and the efficient and stable differentiation of human iPSCs/ESCs into pancreatic endoderm.

## Materials and methods

### Cell lines and maintenance

The experiments using human iPSCs and ESCs were approved by the Ethics Committee of the Department of Medicine and Graduated School of Medicine, Kyoto University. Informed consent was obtained from all donors from whom iPSCs were derived. The iPSC line 585A1^35^ was cultured in Essential 8 medium (Thermo Fisher Scientific). The ESC line KhES-3^36^ and iPSC line Ff-I01^37^ were cultured in StemFit AK02N medium (Reprocell). The cells were maintained under feeder-free conditions, passaged every 3–4 days using 0.5 mM EDTA/PBS (Thermo Fisher Scientific) and gentle pipetting, and routinely examined for mycoplasma contamination.

### Differentiation of iPSCs and ESCs

iPSCs/ESCs were directed to differentiate into pancreatic lineage cells as previously described^38,39^. The medium composition was as follows:

Stage 1 (day 0–3)—RPMI 1640 medium (Nacalai Tesque) supplemented with 2% (vol/vol) B-27 Serum-Free Supplement (B27; Thermo Fisher Scientific), 50 U/mL Penicillin/Streptomycin (P/S; Thermo Fisher Scientific), 100 ng/mL recombinant human/mouse/rat Activin A (R&D Systems) and CHIR99021 (Axon Medchem) at 3 μM on day 0, 1 μM on days 1–2 and 0 μM on day 3.

Stage 2 (day 4–6)—Improved MEM (IMEM) medium (Thermo Fisher Scientific) supplemented with 1% B27, 100 U/mL P/S and 50 ng/mL recombinant human keratinocyte growth factor (KGF; R&D Systems).

Stage 3 (day 7–9)—IMEM medium supplemented with 1% B27, 100 U/mL P/S, 50 ng/mL KGF, 10 nM 4-[(E)-2-(5,6,7,8-Tetrahydro-5,5,8,8-tetramethyl-2-naphthalenyl)-1-propenyl]-benzoic acid (TTNPB; Santa Cruz Biotechnology), 100 ng/mL recombinant human Noggin (PeproTech) and 0.5 μM 3-keto-*N*-aminoethyl-*N*′-aminocaproyldihydrocinnamoyl cyclopamine (KAAD-Cyclopamine; Toronto Research Chemicals).

Stage 4 (day 10–14)—IMEM medium supplemented with 1% B27, 100 U/mL P/S, 100 ng/mL KGF, 50 ng/mL recombinant human epidermal growth factor (EGF; R&D Systems), 10 mM nicotinamide (STEMCELL Technologies), 100 nM (2*S*,5*S*)-(*E,E*)-8-(5-(4-(trifluoromethyl)phenyl)-2,4-pentadienoylamino)benzolactam (TPB; Merck) and 50 μM Y-27632 (Wako).

For continued culture in Stage 4 medium, cell re-seeding was performed on Stage 4 day 4 (day 14) and day 10 (day 20) in the same manner as on Stage 3 day 2 (day 9). For the SHH experiments, Stage 4 medium was supplemented with 50 or 250 ng/mL recombinant human SHH (C24II) (R&D systems) and/or 0.5 μM KAAD-cyclopamine.

For the differentiation of pancreatic β cells, Stage 4 day 4 cells were dissociated into single cells by gentle pipetting after treatment with 0.25% trypsin–EDTA (Invitrogen) for 15 min at 37 ℃, and cell aggregates consisting of 3 × 10^4^ cells were formed in Stage 4 medium using 96-well microplates with cell-repellent surface (Greiner). The following medium changes (200 μL) were done from the next day (day 15) per well every 3 to 4 days:

Stage 5 (day 15–20)—IMEM medium supplemented with 1% B27, 100 U/mL P/S, 1 μM RO4929097 (Selleck), 10 μM Alk 5 inhibitor II (Cayman Chemical), 1 μM Triiodothyronine (Merck Millipore) and 0.1 μM LDN193189 (Axon Medchem).

Stage 6 (day 21–28)—IMEM medium supplemented with 1% B27, 100 U/mL P/S, 10 μM Alk 5 inhibitor II, 1 μM Triiodothyronine and 0.1 μM LDN193189.

### siRNA transfection

Cells were transfected 1 day after seeding on Stage 3 day 2 and differentiated for an additional 4 days. siRNAs were added to the transfection reagent in the Stemfect RNA Transfection Kit (STEMGENT) and incubated for 10 min at room temperature. The transfection mixture was then added to the cells at a final siRNA concentration of 7.5 nM unless otherwise indicated. The transfection efficiency was determined using a BLOCK-iT Alexa Fluor red fluorescent control (Thermo Fisher Scientific), which was transfected into the cells the same way as the siRNA. The siRNAs used are as follows: *PDX1* (siRNA ID s223944; Thermo Fisher Scientific), *ARNTL* (siRNA ID s1616; Thermo Fisher Scientific), *FOXS1* (siRNA ID s5254; Thermo Fisher Scientific), *HHEX* (siRNA ID s6535; Thermo Fisher Scientific), *RUNX3* (siRNA ID s2469; Thermo Fisher Scientific), *SIX4* (siRNA ID s224247; Thermo Fisher Scientific), *SMARCC1* (siRNA ID s13145; Thermo Fisher Scientific), *SMARCD1* (siRNA ID s13152; Thermo Fisher Scientific), and non-targeting control siRNA pool #1 (Catalog ID D-001206-13-05; Horizon Discovery).

### siRNA screening

Posterior foregut cells were seeded on Matrigel (Corning)-coated 96 well plates at 1.6 × 10^5^ cells/cm^2^ one day prior to the addition of siRNA. On the day of the transfection, the medium was replaced with 100 μL of Stage 4 medium using a Biomek NX (Beckman Coulter). A manually aliquoted siGENOME SMARTpool siRNA library (2.5 μM, 2 μL; Horizon Discovery) was diluted using the Stemfect RNA Transfection Kit according to the manufacturer’s instructions. The siRNA transfection complex solution was mixed with 100 μL of Stage 4 medium and then added to the wells. The final concentration of the siRNA was approximately 25 nM. The medium of the wells containing no siRNA or siRNA against *PDX1* and transfection reagent was manually replaced. Four days after the transfection, the cells were fixed and analyzed for a proportion of pancreatic endoderm cells using anti-NKX6.1 antibody (DSHB) staining and cell counting using Hoechst33342 staining (Thermo Fisher Scientific). siRNA against *PDX1* was used as a positive control.

### Immunofluorescence staining

Cells were washed with PBS (Nacalai Tesque) twice, fixed with 4% PFA (Nacalai Tesque)/PBS for 20 min at 4 °C and blocked in PBS with 5% donkey serum (Millipore) and 0.4% Triton X-100 (Nacalai Tesque) (blocking solution) for 30 min at room temperature. The following primary antibodies were diluted in blocking solution and incubated overnight at 4 °C: goat anti-PDX1 (R&D Systems, AF2419, 1:200), mouse anti-NKX6.1 (DSHB, F55A12, 1:200), rabbit anti-NKX6.1 (Cell Signaling, 54551, 1:500) and rat anti-CPEP (DSHB, GN-ID4, 1:200). After washing with PBS, the cells were incubated with the following fluorescent secondary antibodies for 1 h at room temperature: Alexa Fluor 488-conjugated donkey anti-goat (Thermo Fisher Scientific, A-11055, 1:500), Alexa Fluor 546-conjugated donkey anti-mouse (Thermo Fisher Scientific, A-10036, 1:500), Alexa Fluor 555-conjugated donkey anti-rabbit (Thermo Fisher Scientific, A-31572, 1:500) and Alexa Fluor 488-conjugated donkey anti-rat (Thermo Fisher Scientific, A-21208, 1:500). Immunofluorescence images were acquired using a CellInsight NXT (Thermo Fisher Scientific) or BZ-9000 (Keyence). For quantification, 9 images per well were obtained using CellInsight NXT.

### Overexpression

585A1 cells were transfected with a *pCXLE-gw* episomal expression vector encoding *HHEX* (*pCXLE-HHEX*) or *eGFP* (*pCXLE-eGFP*) as a control^40^. 1 × 10^6^ cells were suspended in 100 μL Opti-MEM (Thermo Fisher Scientific), and plasmids were introduced into the cells by electroporation using the Super Electroporator NEPA 21 (NEPA GENE) under the following conditions: (A) pulse voltage, 125 V; pulse interval, 50 ms; pulse length, 5 ms; and pulse number, 2; or (B) pulse voltage, 20 V; pulse interval, 50 ms; pulse length, 50 ms; and pulse number, 5. The cells were cultured in Stage 4 medium for 4 days after transfection. Since multiple plasmids are not independently transfected but easily co-transfected into the cells^41^, we co-transfected iPSCs with *pCXLE-HHEX* and *pCXLE-eGFP* and concentrated *pCXLE-HHEX*-transfected cells by flowcytometry sorting eGFP-positive cells. 7.5 μg *pCXLE-HHEX* and 2.5 μg *pCXLE-eGFP* were introduced for HHEX overexpression, and 10 μg *pCXLE-eGFP* was introduced as a control per 1 × 10^6^ cells.

### Flow cytometry

Cells were dissociated into single cells by gentle pipetting after treatment with 0.25% trypsin–EDTA (Invitrogen) for 15 min at 37 ℃ and fixed with a Cytofix/Cytoperm Kit (BD Biosciences) according to the manufacturer’s protocol. Then, the cells were blocked with 2% donkey serum in permeabilization solution and incubated with the following primary antibodies diluted in blocking solution overnight at 4 °C: goat anti-PDX1 (R&D Systems, AF2419, 1:200), mouse anti-NKX6.1 (DSHB, F55A12, 1:200), rabbit anti-NKX6.1 (Cell Signaling, 54551, 1:500) and rat anti-CPEP (DSHB, GN-ID4, 1:200). After washing with wash buffer once, the cells were incubated with the following fluorescent secondary antibodies for an hour at room temperature: Alexa Fluor 488-conjugated donkey anti-goat (Thermo Fisher Scientific, A-11055, 1:500), Alexa Fluor 647-conjugated donkey anti-mouse (Thermo Fisher Scientific, A-31571, 1:500), Alexa Fluor 647-conjugated donkey anti-rabbit (Thermo Fisher Scientific, A-31573, 1:500) and Alexa Fluor 488-conjugated donkey anti-rat (Thermo Fisher Scientific, A-21208, 1:500). The stained cells were analyzed using a FACSAria II or LSRFortessa (BD Biosciences). The cell number was counted using a TC20™ automatic cell counter (Bio-Rad).

### Western blotting

Cells were collected and lysed with RIPA Buffer (Wako) containing 1% protease inhibitor cocktail (Sigma-Aldrich). The lysate was mixed with Sample Buffer Solution with 2-mercaptoethanol (Nacalai Tesque), incubated for 3 min at 95 °C, loaded into a 4–20% Mini-protein TGX precast gel (Bio-Rad) and transferred to a PVDF membrane using a Trans-Blot Turbo Transfer System (Bio-Rad) after electrophoresis. Then, the membrane was blocked with 5% skim milk in Tris-based saline with Tween 20 (0.1% TBS-T) buffer and incubated with the following primary antibodies diluted in blocking solution overnight at 4 °C: rabbit anti-HHEX (R&D Systems, MAB83771-100, 1:200), goat anti-PDX1 (R&D Systems, AF2419, 1:5000), rabbit anti-NKX6.1 (Cell Signaling, 54551, 1:1000) and mouse anti-GAPDH (Sigma-Aldrich, MAB374, 1:300). After washing with TBS-T, the membrane was incubated with the following secondary antibodies diluted in blocking solution for an hour at room temperature: horseradish peroxidase (HRP) conjugated sheep anti-mouse (Cytiva, NA931VS, 1:3000), HRP conjugated donkey anti-rabbit (Cytiva, NA934VS, 1:3000) and HRP conjugated donkey anti-goat (Abcam, ab6885, 1:5000). After washing with TBS-T, the membrane was incubated with ECL Western Blotting Detection Reagent (Amersham) for 5 min at room temperature. The protein bands were visualized using an ImageQuant 800 (Cytiva).

### Real-time quantitative reverse transcription polymerase chain reaction (qRT-PCR)

Total RNA was isolated from the cells with the RNeasy Mini Kit (Qiagen) according to the manufacturer’s instructions and reverse transcribed using ReverTra Ace qPCR Master Mix (TOYOBO), dNTP Mix (Qiagen) and oligo dT primer (FASMAC). SYBR Premix Ex Taq II (Takara) was used for quantitative PCR on a StepOnePlus Real-Time PCR System (Applied Biosystems). Each expression level of the target genes was normalized to those of *beta-actin* (*ACTB*). The primer sequences were as follows: *ACTB*, 5′-CATGTACGTTGCTATCCAGGC-3′ and 5′-CTCCTTAATGTCACGCACGAT-3′; *HHEX*, 5′-CACCCGACGCCCTTTTACAT-3′ and 5′-GAAGGCTGGATGGATCGGC-3′; *PDX1*, 5′-AGCAGTGCAAGAGTCCCTGT-3′ and 5′-CACAGCCTCTACCTCGGAAC-3′; *NKX6.1*, 5′-ATTCGTTGGGGATGACAGAG-3′ and 5′-TGGGATCCAGAGGCTTATTG-3′; *PTF1A*, 5′-CCCCAGCGACCCTGATTA-3′ and 5′-GGACACAAACTCAAATGGTGG-3′; *ONECUT1*, 5′-AACCCTGGAGCAAACTCAAA-3′ and 5′-TGGATGGACGCTTATTTTCC-3′; *ONECUT3*, 5′-GGCCCAGTGAGCTTCTTAGA-3′ and 5′-GAACGATCCCAAGCCAAGTC-3′; and *EPCAM*, 5′-CGCAGCTCAGGAAGAATGTG-3′ and 5′-TGAAGTACACTGGCATTGACG-3′.

### RNA sequencing

Total RNA was isolated from the cells with the RNeasy Mini Kit according to the manufacturer’s instructions. The samples were preserved at −80 °C before use. The RNA sequence library preparation, sequencing, mapping and gene expression analysis were performed by DNAFORM (Yokohama, Japan). The quality of total RNA was assessed using a Bioanalyzer (Agilent) to ensure that the RNA integrity number (RIN) was over 7.0. After poly (A) + RNA enrichment using a NEBNext Poly (A) mRNA Magnetic Isolation Module (New England BioLabs), double-stranded cDNA libraries (RNA-seq libraries) were prepared using a SMART Seq^®^ Stranded Kit (TaKaRa) according to the manufacturer’s instructions. RNA-seq libraries were sequenced using paired end reads (50 nt of read 1 and 25 nt of read 2) on a NextSeq 500 instrument (Illumina). Obtained raw reads were trimmed and quality-filtered using the Trim Galore! (version 0.6.6), Trimmomatic (version 0.39), and cutadapt (version 3.1) software. Trimmed reads were mapped to the human GRCh38 genome using STAR (version 2.7.3a). Reads on annotated genes were counted using featureCounts (version 2.0.1). TPM values were calculated based on FPKM values, and heatmaps were created by converting them into normalized Z-scores for each gene. The gene sets of pancreas, hepatobiliary, duodenum primordium and definitive endoderm markers are provided with reference to previous reports^42–44^.

### Statistics

All quantitative data are presented as the mean ± SD. Data were analyzed for statistical significance using Microsoft Excel for Windows version 2202 and R version 4.1.2 (R Core Team (2021). R: A language and environment for statistical computing. R Foundation for Statistical Computing, Vienna, Austria, http://www.R-project.org/). Two-tailed Student’s t-test was used to compare the means of two groups, and one-way ANOVA with Tukey or Dunnett’s test was used for multiple comparisons between groups. *p* < 0.05 was considered statistically significant for all analyses. *p < 0.05 and **p < 0.01 in the figures.

## Supplementary Information

Supplementary Figures.

## Data Availability

The datasets used and/or analysed during the current study are available from the corresponding author upon reasonable request. The NCBI GEO accession number for RNA sequencing data reported in this paper is GSE 222068.

## References

[1] DiMeglio LA, Evans-Molina C, Oram RA (2018). Type 1 diabetes. Lancet.

[2] Shapiro AMJ, Pokrywczynska M, Ricordi C (2017). Clinical pancreatic islet transplantation. Nat. Rev. Endocrinol..

[3] Nakamura T (2020). Long-term outcome of islet transplantation on insulin-dependent diabetes mellitus: An observational cohort study. J. Diabetes Investig..

[4] D'Amour KA (2006). Production of pancreatic hormone-expressing endocrine cells from human embryonic stem cells. Nat. Biotechnol..

[5] Kroon E (2008). Pancreatic endoderm derived from human embryonic stem cells generates glucose-responsive insulin-secreting cells in vivo. Nat. Biotechnol..

[6] Pagliuca FW (2014). Generation of functional human pancreatic β cells in vitro. Cell.

[7] Rezania A (2014). Reversal of diabetes with insulin-producing cells derived in vitro from human pluripotent stem cells. Nat. Biotechnol..

[8] Russ HA (2015). Controlled induction of human pancreatic progenitors produces functional beta-like cells in vitro. EMBO J..

[9] Toyoda T (2015). Cell aggregation optimizes the differentiation of human ESCs and iPSCs into pancreatic bud-like progenitor cells. Stem Cell Res..

[10] Toyoda T (2017). Rho-associated kinases and non-muscle myosin IIs inhibit the differentiation of human iPSCs to pancreatic endoderm. Stem Cell Rep..

[11] Jennings RE (2013). Development of the human pancreas from foregut to endocrine commitment. Diabetes.

[12] Aigha II, Abdelalim EM (2020). NKX6.1 transcription factor: A crucial regulator of pancreatic β cell development, identity, and proliferation. Stem Cell Res. Ther..

[13] Rezania A (2013). Enrichment of human embryonic stem cell-derived NKX6.1-expressing pancreatic progenitor cells accelerates the maturation of insulin-secreting cells in vivo. Stem Cells.

[14] Pedersen JK (2005). Beta cell biology consortium, endodermal expression of Nkx6 genes depends differentially on Pdx1. Dev. Biol..

[15] Camp JG (2017). Multilineage communication regulates human liver bud development from pluripotency. Nature.

[16] Shi Z-D (2017). Genome editing in hPSCs reveals GATA6 haploinsufficiency and a genetic interaction with GATA4 in human pancreatic development. Cell Stem Cell.

[17] Martinez-Barbera JP (2000). The homeobox gene Hex is required in definitive endodermal tissues for normal forebrain, liver and thyroid formation. Development.

[18] Bogue CW, Ganea GR, Sturm E, Ianucci R, Jacobs HC (2000). Hex expression suggests a role in the development and function of organs derived from foregut endoderm. Dev. Dyn..

[19] Bort R, Signore M, Tremblay K, Martinez-Barbera JP, Zaret KS (2006). Hex homeobox gene controls the transition of the endoderm to a pseudostratified, cell emergent epithelium for liver bud development. Dev. Biol..

[20] Kimura A, Toyoda T, Iwasaki M, Hirama R, Osafune K (2020). Combined omics approaches reveal the roles of non-canonical WNT7B signaling and YY1 in the proliferation of human pancreatic progenitor cells. Cell Chem. Biol..

[21] Larribère L (2017). An RNAi screen reveals an essential role for HIPK4 in human skin epithelial differentiation from iPSCs. Stem Cell Rep..

[22] Thomas PQ, Brown A, Beddington RS (1998). Hex: A homeobox gene revealing peri-implantation asymmetry in the mouse embryo and an early transient marker of endothelial cell precursors. Development.

[23] Soufi A, Jayaraman P-S (2008). PRH/Hex: An oligomeric transcription factor and multifunctional regulator of cell fate. Biochem. J..

[24] Bort R, Martinez-Barbera JP, Beddington RSP, Zaret KS (2004). Hex homeobox gene-dependent tissue positioning is required for organogenesis of the ventral pancreas. Development.

[25] Yang D (2022). CRISPR screening uncovers a central requirement for HHEX in pancreatic lineage commitment and plasticity restriction. Nat. Cell Biol..

[26] Zhang J, McKenna LB, Bogue CW, Kaestner KH (2014). The diabetes gene Hhex maintains δ-cell differentiation and islet function. Genes. Dev..

[27] Sladek R (2007). A genome-wide association study identifies novel risk loci for type 2 diabetes. Nature.

[28] Horikoshi M (2007). Variations in the HHEX gene are associated with increased risk of type 2 diabetes in the Japanese population. Diabetologia.

[29] Vliet-Ostaptchouk JV (2008). HHEX gene polymorphisms are associated with type 2 diabetes in the Dutch Breda cohort. Eur. J. Hum. Genet..

[30] Heller S (2021). Transcriptional changes and the role of ONECUT1 in hPSC pancreatic differentiation. Commun. Biol..

[31] Pierreux CE, Vanhorenbeeck V, Jacquemin P, Lemaigre FP, Rousseau GG (2004). The transcription factor hepatocyte nuclear factor-6/Onecut-1 controls the expression of its paralog Onecut-3 in developing mouse endoderm. J. Biol. Chem..

[32] Vanhorenbeeck V (2007). Role of the Onecut transcription factors in pancreas morphogenesis and in pancreatic and enteric endocrine differentiation. Dev. Biol..

[33] Watanabe H (2014). HHEX promotes hepatic-lineage specification through the negative regulation of eomesodermin. PLoS ONE.

[34] Paz H, Lynch MR, Bogue CW, Gasson JC (2010). The homeobox gene Hhex regulates the earliest stages of definitive hematopoiesis. Blood.

[35] Okita K (2013). An efficient nonviral method to generate integration-free human-induced pluripotent stem cells from cord blood and peripheral blood cells. Stem Cells.

[36] Suemori H (2006). Efficient establishment of human embryonic stem cell lines and long-term maintenance with stable karyotype by enzymatic bulk passage. Biochem. Biophys. Res. Commun..

[37] Nakagawa M (2014). A novel efficient feeder-free culture system for the derivation of human induced pluripotent stem cells. Sci. Rep..

[38] Kimura A (2017). Small molecule AT7867 proliferates PDX1-expressing pancreatic progenitor cells derived from human pluripotent stem cells. Stem Cell Res..

[39] Tsujisaka Y (2022). Purification of human iPSC-derived cells at large scale using microRNA switch and magnetic-activated cell sorting. Stem Cell Rep..

[40] Yamakawa T (2016). Screening of human cDNA library reveals two differentiation-related genes, HHEX and HLX, as promoters of early phase reprogramming toward pluripotency. Stem Cells.

[41] Hannig G, Jany C (2013). Co-transfection of plasmid DNA. Biotechniques.

[42] Koike H (2019). Modelling human hepato-biliary-pancreatic organogenesis from the foregut–midgut boundary. Nature.

[43] Tsai Y-H (2017). In vitro patterning of pluripotent stem cell-derived intestine recapitulates in vivo human development. Development.

[44] Hung Y-H (2021). Chromatin regulatory dynamics of early human small intestinal development using a directed differentiation model. Nucleic Acids Res..

