# Supplementary material for: Elucidation of HHEX in pancreatic endoderm differentiation using a human iPSC differentiation model
Source: Sci Rep. 2023 May 29;13:8659. doi: 10.1038/s41598-023-35875-1 (PMC10227077; doi:10.1038/s41598-023-35875-1)

Supplementary Information

Elucidation of HHEX in pancreatic endoderm differentiation using a human iPSC differentiation model

Ryo Ito[1, 2], Azuma Kimura[1], Yurie Hirose[1], Yu Hatano[1], Atsushi Mima[1], Shin-Ichi Mae[1], Yamato Keidai[2], Toshihiro Nakamura[2], Junji Fujikura[2], Yohei Nishi[1], Akira Ohta[1], Taro Toyoda[1*], Nobuya Inagaki[2], Kenji Osafune[1*]

1. Center for iPS Cell Research and Application (CiRA), Kyoto University, 53 Kawahara-cho, Shogoin, Sakyo-ku, Kyoto, 606-8507, Japan.
2. Department of Diabetes, Endocrinology and Nutrition, Graduate School of Medicine, Kyoto University, 54 Kawahara-cho, Shogoin, Sakyo-ku, Kyoto, 606-8507, Japan.

*Correspondence should be addressed to T.T. (e-mail address: t.toyoda@cira.kyoto-u.ac.jp, fax number: +81-75-366-7023) and K.O. (e-mail address: osafu@cira.kyoto-u.ac.jp, fax number: +81-75-366-7077).

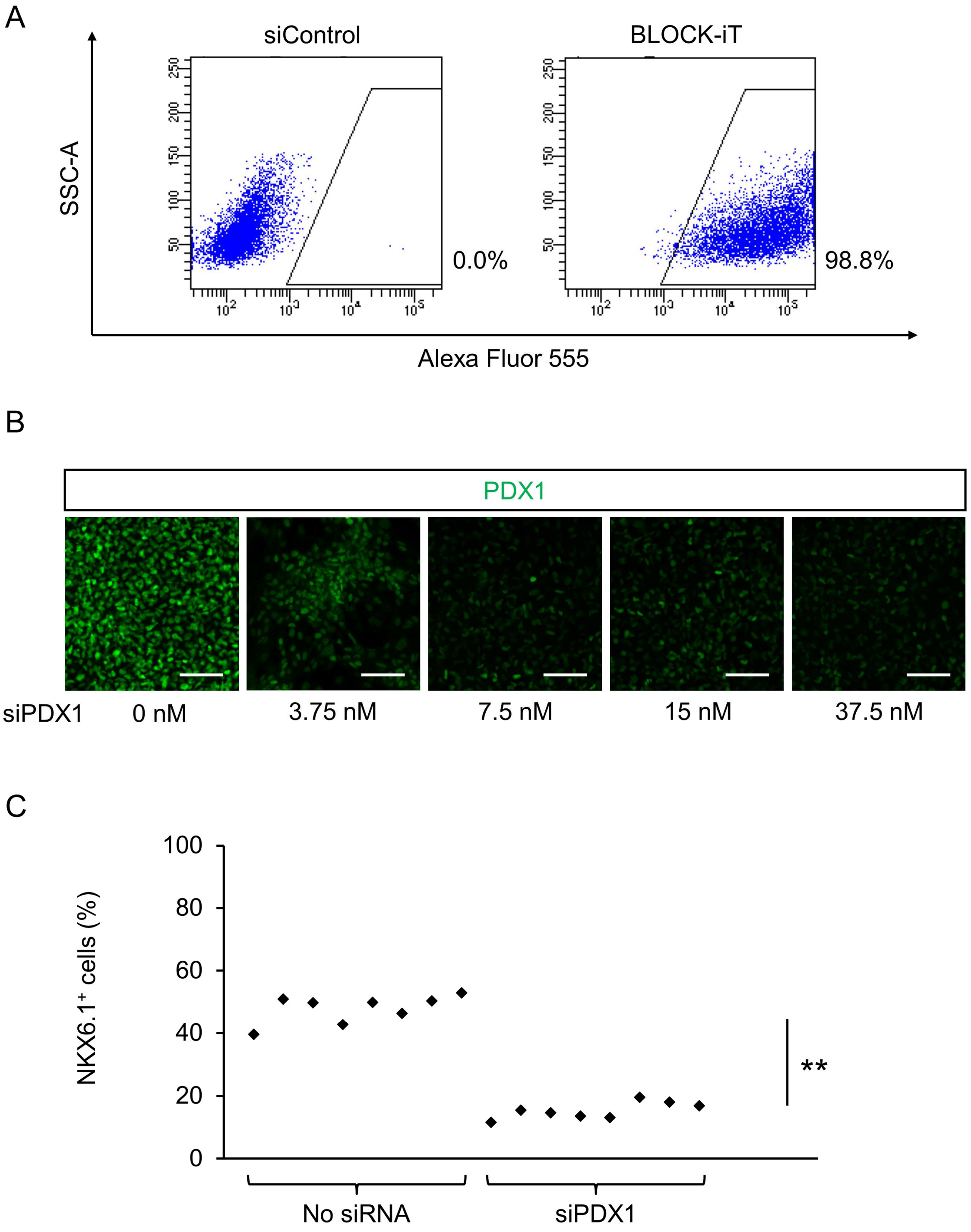
A
siControl
BLOCK-iT
SSC-A
0.0%
98.8%
Alexa Fluor 555
B
PDX1
siPDX1
0 nM
3.75 nM
7.5 nM
15 nM
37.5 nM
C
NKX6.1+ cells (%)
100
80
60
40
20
0
**
No siRNA
siPDX1

Figure S1. siRNA knockdown screening system for regulators of NKX6.1 expression in pancreatic endoderm.

(A) Representative flow cytometer plots showing the transfection efficiency of BLOCK-iT Alexa Fluor red fluorescent control into pancreatic endoderm cells. **(B)** Immunofluorescence images of pancreatic endoderm cells transfected with various concentrations of siPDX1 for PDX1. Scale bars, 100 μm. **(C)** Scatterplot distribution of NKX6.1^{+} cell percentage per well with or without PDX1 siRNA. $^{**}p < 0.01$, two-tailed Student's t-test.

A

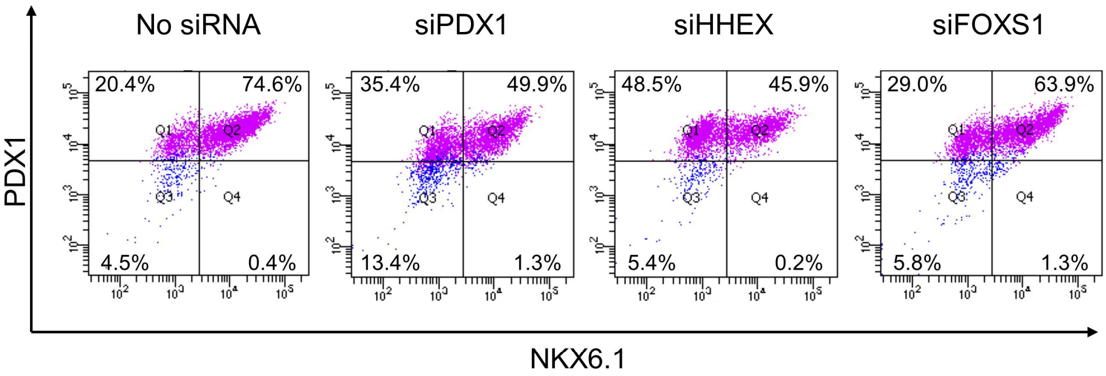
No siRNA
siPDX1
siHHEX
siFOXS1
20.4%
74.6%
4.5%
0.4%
35.4%
49.9%
13.4%
1.3%
48.5%
45.9%
5.4%
0.2%
29.0%
63.9%
5.8%
1.3%
Q1
Q2
Q3
Q4
PDX1
NKX6.1

B

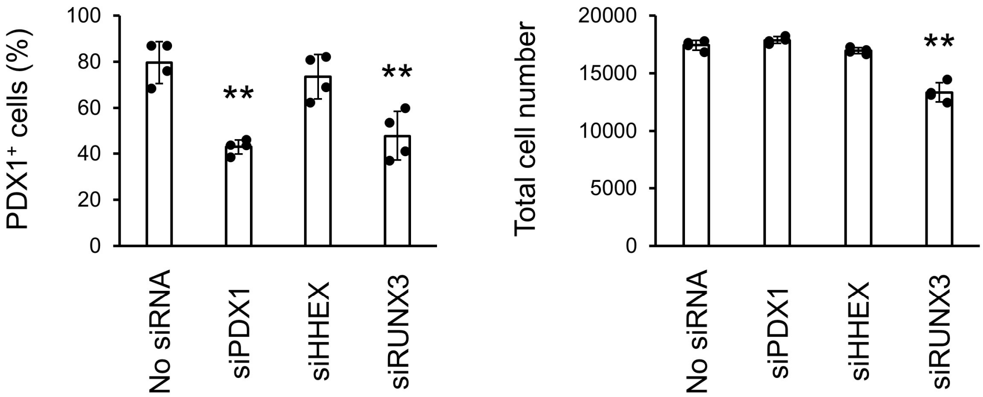
PDX1+ cells (%)
100
80
60
40
20
0
**
**
No siRNA
siPDX1
siHHEX
siRUNX3
Total cell number
20000
15000
10000
5000
0
**
No siRNA
siPDX1
siHHEX
siRUNX3

Figure S2. Reproducibility of siRNA screening for regulators of NKX6.1 expression in pancreatic endoderm.

(A) Representative flow cytometry dot plots and percentages of $PDX1^-NKX6.1^-$, $PDX1^+NKX6.1^-$, $PDX1^-NKX6.1^+$ and $PDX1^+NKX6.1^+$ cells upon knockdown of *PDX1*, *HHEX* or *FOXS1*. **(B)** Percentage of $PDX1^+$ cells (left panel) and total cell number (right panel) upon knockdown of *PDX1*, *HHEX* or *RUNX3* as evaluated by immunostaining image analysis. The data from four independent experiments are presented as the mean ± SD (n=4). **$p < 0.01$ by one-way ANOVA with Dunnett's test for comparison with no siRNA.

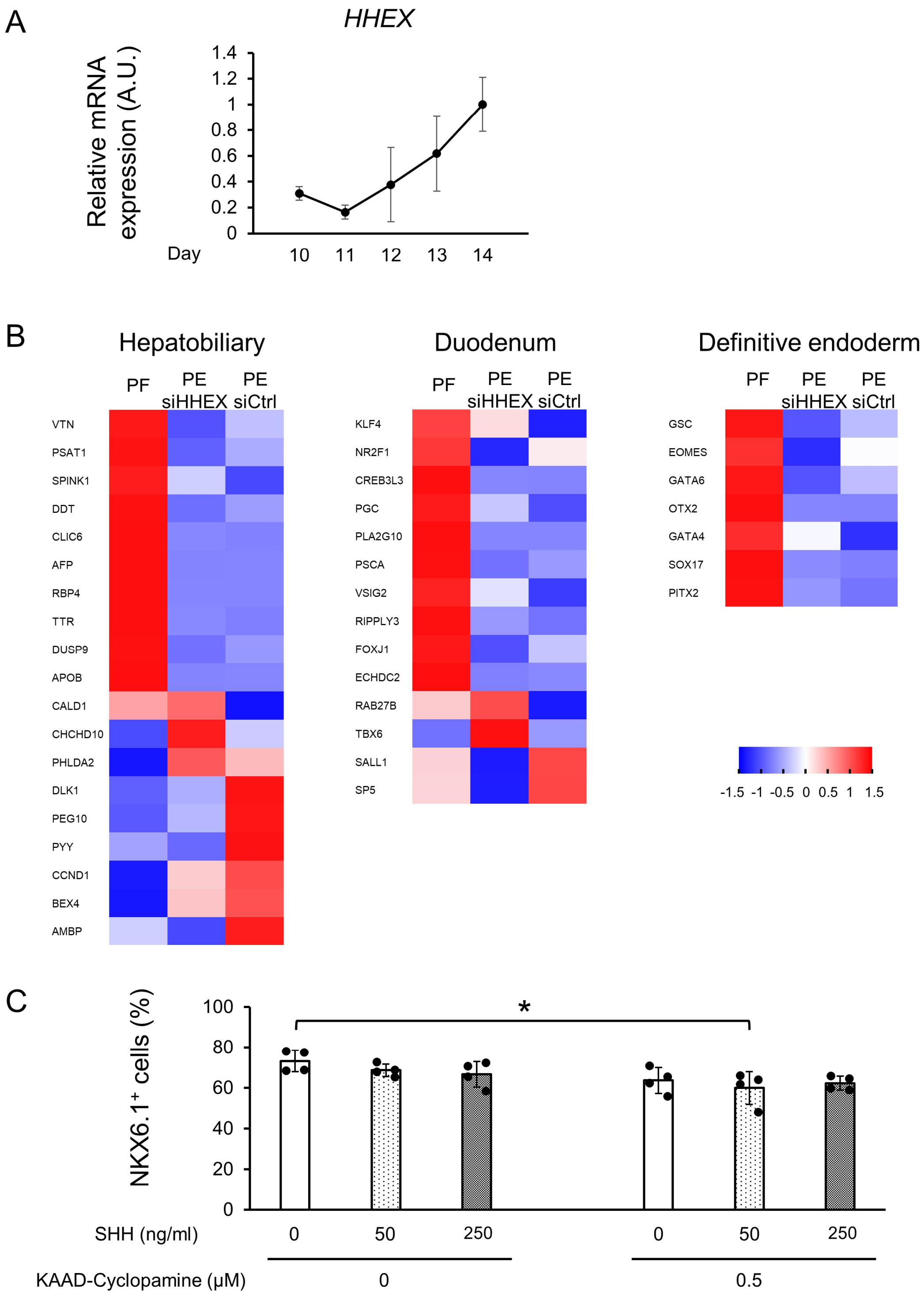

A
HHEX
Relative mRNA expression (A.U.)
1.4
1.2
1
0.8
0.6
0.4
0.2
0
Day
10
11
12
13
14
B
Hepatobiliary
PF
PE siHHEX
PE siCtrl
VTN
PSAT1
SPINK1
DDT
CLIC6
AFP
RBP4
TTR
DUSP9
APOB
CALD1
CHCHD10
PHLDA2
DLK1
PEG10
PYY
CCND1
BEX4
AMBP
Duodenum
PF
PE siHHEX
PE siCtrl
KLF4
NR2F1
CREB3L3
PGC
PLA2G10
PSCA
VSIG2
RIPPLY3
FOXJ1
ECHDC2
RAB27B
TBX6
SALL1
SP5
Definitive endoderm
PF
PE siHHEX
PE siCtrl
GSC
EOMES
GATA6
OTX2
GATA4
SOX17
PITX2
-1.5 -1 -0.5 0 0.5 1 1.5
C
NKX6.1+ cells (%)
100
80
60
40
20
0
*
SHH (ng/ml)
0
50
250
0
50
250
KAAD-Cyclopamine (μM)
0
0.5

Figure S3. Verification of HHEX in pancreatic endoderm differentiation.
(A) *HHEX* expression during differentiation from posterior foregut to pancreatic endoderm using modified Stage 4 medium without EGF, nicotinamide or TPB. **(B)** Heatmaps representing normalized Z-scores of hepatobiliary, duodenum and definitive endoderm markers in posterior foregut (PF) and pancreatic endoderm (PE) with or without *HHEX* knockdown. **(C)** Percentage of NKX6.1$^+$ cells Stage 4 upon the addition of Sonic Hedgehog (SHH) and/or KAAD-Cyclopamine. The data from four independent experiments are presented as the mean ± SD (n=4). *$p < 0.05$ by one-way ANOVA with Tukey's test.

Full unedited membrane view for Figure 2B

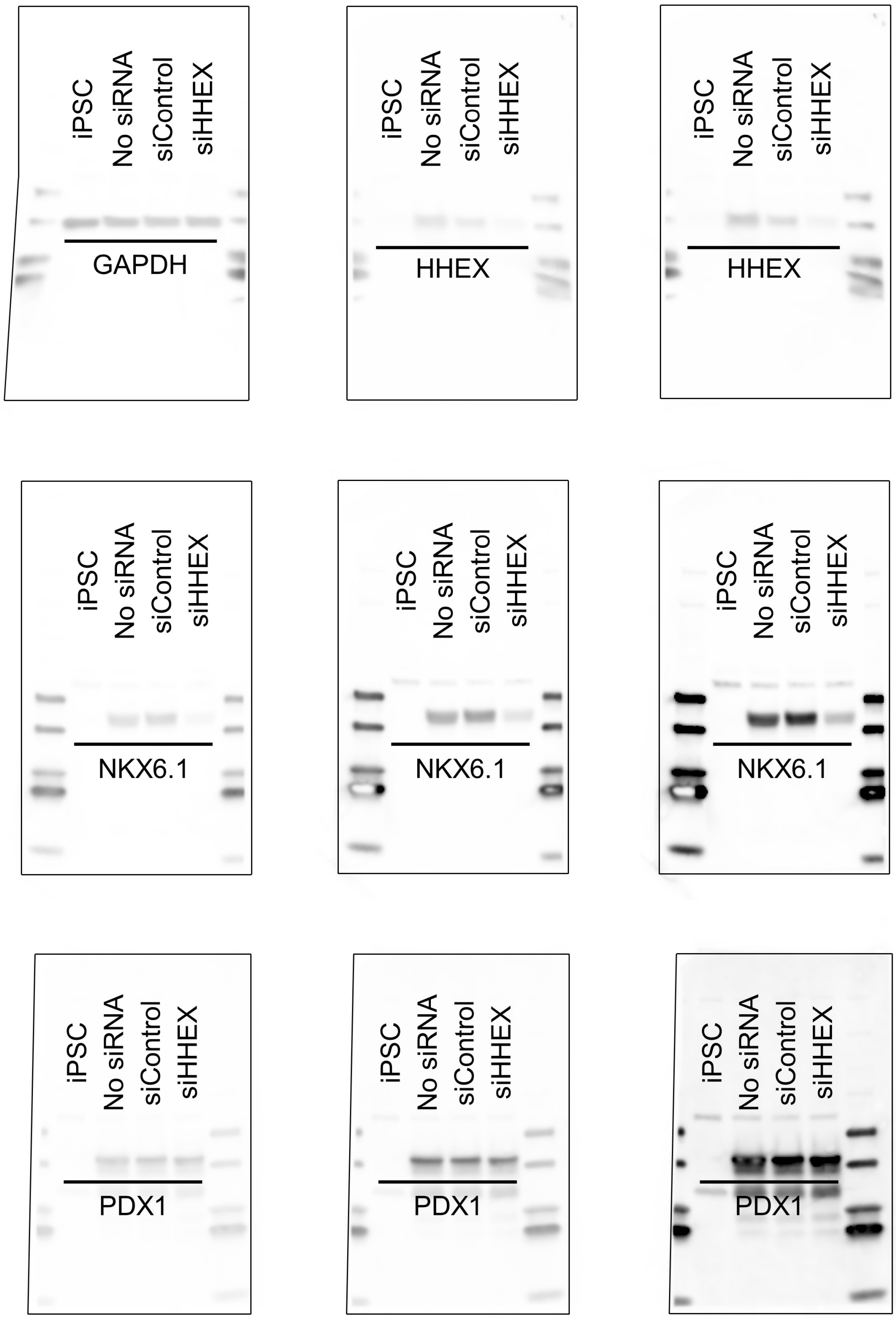

Supplement: Supplementary file 1 — Supplementary Figures. [file 41598_2023_35875_MOESM1_ESM.pdf]
